# A Simple “Blood-Saving Bundle” Reduces Diagnostic Blood Loss and the Transfusion Rate in Mechanically Ventilated Patients

**DOI:** 10.1371/journal.pone.0138879

**Published:** 2015-09-30

**Authors:** Reimer Riessen, Melanie Behmenburg, Gunnar Blumenstock, Doris Guenon, Sigrid Enkel, Richard Schäfer, Michael Haap

**Affiliations:** 1 Department of Internal Medicine, Medical Intensive Care Unit, University of Tübingen, Tübingen, Germany; 2 Department of Clinical Epidemiology and Applied Biometry, University of Tübingen, Tübingen, Germany; 3 Clinical Transfusion Medicine, University of Tübingen, Tübingen, Germany; 4 Institute for Transfusion Medicine and Immunohaematology, German Red Cross Blood Donor Service Baden-Württemberg-Hessen GmbH, Johann-Wolfgang-Goethe-University Hospital, Frankfurt/Main, Germany; Azienda Ospedaliero-Universitaria Careggi, ITALY

## Abstract

**Introduction:**

Aim of this study was to reduce blood loss caused by diagnostic blood sampling and to minimize the development of anemia in a high-risk group of mechanically ventilated medical intensive care patients. We therefore implemented a “blood-saving bundle” (BSB) combining a closed-loop arterial blood sampling system, smaller sampling tubes, reduced frequency of blood drawings, and reduced sample numbers.

**Methods:**

The study included all patients from our medical ICU who were ventilated for more than 72 hours. Exclusion criteria were: acute or chronic anemia on admission, bleeding episode(s) during the ICU stay, or end-of-life therapy. The BSB was introduced in 2009 with training and educational support. Patients treated in 2008, before the introduction of the BSB, served as a control group (n = 41, 617 observation days), and were compared with patients treated in 2010 after the introduction of the BSB (BSB group, n = 50, 559 observation days). Primary endpoints were blood loss per day, and development of anemia. Secondary endpoints were numbers of blood transfusions, number of days on mechanical ventilation, and length of the ICU stay.

**Results:**

Mean blood loss per ICU day was decreased from 43.3 ml (95% CI: 41.2 to 45.3 ml) in the controls to 15.0 ml (14.3 to 15.7 ml) in the BSB group (P < 0.001). The introduction of a closed-loop arterial blood sampling system was the major contributor to this effect. Mean hemoglobin concentrations showed no significant differences in both groups during the ICU stay. Hemoglobin values <9 g/dl, however, were recorded in 21.2% of observation days in the controls versus 15.4% in the BSB group (P = 0.01). Units of transfused red blood cells per 100 observation days decreased from 7 to 2.3 (P < 0.001). The mean number of ventilation days was 7.1 days (6.1 to 8.3 days) in the controls and 7.5 days (6.6 to 8.5 days) in the BSB group (P = NS). In total, patients in the BSB group stayed in ICU for a mean of 9.9 days (8.6 to 11.3 days), compared to a mean ICU stay of 13.0 days (10.9 to 15.4 days) in the control group (P = 0.014). Due to the longitudinal study design, however, we cannot exclude uncontrolled confounders affecting the transfusion frequency and mean ICU stay.

**Conclusion:**

Our BSB could be easily implemented and was able to reduce diagnostic blood loss.

## Introduction

The majority of critically ill patients develops an anemia after several days in the intensive care unit (ICU) [1,2]. The causes for the anemia in these patients are multifactorial. Erythropoiesis can be impaired by inflammation, the release of cytokines, changes in iron metabolism as well as by reduced release and action of erythropoietin [3]. Other contributing factors can be an overt or occult blood loss through bleeding or a reduced red blood cell (RBC) life span [4]. Another, at least partially preventable cause is diagnostic blood loss due to phlebotomy and repetitive laboratory testing [5]. As reported in several larger studies [1,4–6], diagnostic blood loss of 40–50 ml per ICU day could contribute to up to 20% of the total blood loss during an ICU stay leading to anemia [4]. Notably, after one week in the ICU, this diagnostic blood loss is equivalent to the volume of one unit of packed RBCs. Studies in Europe and Northern America have shown that 39–44% of all ICU patients do receive RBC transfusions, and this rate increases with the duration of the ICU stay [1,2,7]. In the *Anemia and blood transfusion in critically ill patients* (ABC) study, patients with an ICU stay longer than 7 days had a RBC transfusion rate of 73% [1]. In the *Audit of Transfusion in Intensive Care in Scotland* (ATICS) study, 60% of all transfused RBC units were given to patients without clinically significant bleeding signs. These patients received on average 1.9 units of RBCs per transfusion episode. From a pathophysiological standpoint, anemia can reduce oxygen delivery especially in patients with cardiac and respiratory disease and is associated with a negative clinical outcome [8]. However, there is no clear evidence that a liberal blood transfusion policy in moderate anemia can improve outcome. Blood transfusions are associated with a substantial number of infectious and non-infectious serious hazards of transfusion, such as transfusion-related acute lung injury, transfusion-related immunomodulation, alloimmunization, metabolic derangements, transfusion-associated circulatory overload, post-transfusion purpura, transfusion-associated graft versus host disease, and complications from red cell storage lesions [9,10].

Therefore, most guidelines recommend a hemoglobin concentration of 6–8 g/dl as a transfusion trigger in patients with acute blood loss and critically ill patients with anemia [11].

Taken this together, it seems reasonable to attack all preventable causes of anemia in critically ill patients in order to reduce unnecessary blood transfusions and transfusion associated side effects [12]. Since the early 1990s, several studies have attempted to reduce diagnostic blood loss, mainly by using blood conservation devices, which minimize the amount of discarded blood when samples are taken from arterial or central lines [5]. However, only one recent study could show an effect on anemia and blood transfusions in a relatively unselected group on intensive care patients [13]. Another approach is the use of smaller-size blood collection tubes, which also have been shown to reduce daily blood loss [14]. In our study we combined several approaches in a so-called “blood-saving bundle” (BSB):

1. Blood conservation devices for arterial lines
2. Reduction of the size of the blood collection tubes
3. Policy to use non-invasive methods such as pulse oxymetry and capnography as often as possible to adjust mechanical ventilation in order to reduce the frequency of invasive arterial blood gas analyses.

We tested this approach in a well-defined high-risk group of intensive care patients on prolonged mechanical ventilation using a longitudinal before-and-after study design.

## Material and Methods

### Study Design

This was a before-and-after study in a 21 bed medical intensive care and intermediate care unit of a university hospital. Patients treated in 2008 served as the control group. In 2009 the BSB was introduced. For the intervention group (“BSB-group”) we analyzed patients treated in 2010, when the BSB was in routine use. The study was approved by the Ethics committee of the University of Tübingen. To carry out the study, no written consent was needed.

### Study Population

During the observation period, all patients treated with mechanical ventilation for longer than 72 h and without any exclusion criteria were included into the study.

Exclusion criteria were:

- Moderate anemia (Hb < 9 g/dl) on admission
- History of a chronic hereditary or acquired hematological disease with anemia
- Chronic renal failure (stage G4 and G5), renal anemia and/or treatment with erythropoietin
- Acute clinically relevant bleeding or decrease of the hemoglobin concentration of ≥3 g/dl during the first 24 h after admission
- Transfusion requirement of ≥ 3 RBCs within one week in the last 4 weeks before admission
- End-of-life therapy or death of the patient

### Blood Saving Bundle (BSB)

In 2009 the BSB was introduced and applied to all patients in the ICU. The BSB comprised the following components:

- Arterial blood conservation device: Before the introduction of the BSB arterial blood samples were taken from a three way stopcock. To clear the line from flushing solution, first a serum tube was connected to the stopcock, and several ml of blood were drawn into the serum tube before the blood sample was taken. The serum tube was then reconnected, the stopcock cleared with flushing solution, and the serum tube was discarded. After testing devices from different vendors, an arterial blood conservation device (xtrans^®^ ABSS, Kodan pvb, Lensahn, Germany) was introduced. Before blood samples were taken from a port integrated into the arterial line, blood and flush solution were drawn into a reservoir distal to this sampling port. After taking the sample the blood that was preserved in the reservoir was re-infused. Hereby, no blood was discarded.
- Nurses used smaller sample volumes (approx. 1 ml) for arterial blood gas tests and other diagnostic samples, if technically possible.
- The size of the blood collection tubes (Sarstedt Monovette^®^, Nümbrecht, Germany) was reviewed. A switch to pediatric collection tubes was deemed impractical, because these tubes were not compatible with the laboratory analyzers. The size of the standard lithium-heparin serum tube could be reduced from 5.5 ml to 2.7 ml. The size of all other collection tubes could not be reduced.
- A policy was established to use pulse oxymetry and capnography as often as possible to monitor oxygenation and ventilation in order to reduce the frequency of arterial blood gas analyses with the point-of-care analyzer. These samples, however, were also used to measure Hb-levels, electrolytes, lactate and blood glucose to guide several aspects of ICU-therapy in the critically ill patients included in this study and were drawn by nurses or residents when they deemed it necessary for clinical decision making. For isolated measurements of blood glucose in stable patients we encouraged the use of glucose sticks with a handheld analyzer (Akku-Chek^®^ performa, Roche, Mannheim, Germany).

The ICU staff training program was designed as follows: During the introduction phase short presentations about the BSB were given to the nurses during handovers. Nurses were also supplied with printed educational material and were coached individually at the bedside by one member of the study team (M.B.).

### Blood Transfusions

Physicians were instructed in both study periods to apply a standard transfusion Hb-threshold of 7 g/dl. In patients with cardiovascular or respiratory instability, a transfusion threshold of 8 or 9 g/dl was allowed on an individual basis usually after discussion on rounds.

### Data Collection

In 2008 and 2010 representative numbers (n = 50 or n = 100) of all blood samples and discarded blood volumes were collected and analyzed in a standardized protocol in order to calculate mean blood volume associated with every type of blood sampling as a reference for the calculation of the blood loss (see below).

All patients treated with mechanical ventilation for > 72 h in 2008 and 2010 were selected from a data base and checked for exclusion criteria.

The electronic medical records of all included patients were analyzed to determine the number of all blood sampling events and the corresponding number of all collection and flushing tubes including samples for blood cultures and blood compatibility tests. These numbers were multiplied with the reference blood volume for each type of tube in order to calculate the resulting blood loss.

For the analysis of hemoglobin concentrations we used the values measured at admission and the follow-up values measured daily at 5:00 a.m. in the central laboratory. We also recorded the Hb levels that triggered RBC transfusions. All other data such as patient characteristics, Simplified Acute Physiology Score II (SAPS II), numbers of transfused RBC units, length of ICU stay, and duration of mechanical ventilation were also retrieved from the electronic medical records.

### Primary Endpoint

Mean daily blood loss (per ICU day, ventilation day, non-ventilation day) Development of anemia

### Secondary Endpoints

Daily change of the hemoglobin concentration

Number of observation days with hemoglobin concentrations < 9 g/dl and < 8 g/dl Blood transfusions (number of transfusion events, number of RBC units) ICU length of stay

Duration of mechanical ventilation

### Sample Size

With 90% power and a two-sided two group t test, a sample size of 39 patients in each of the groups will detect an effect of at least δ = 0.75. This means for instance, in concrete terms, a relevant reduction of 20% from a before mean blood loss of 45 ml per day assuming a SD of 12 ml (nQuery Advisor 4.0, Statistical Solutions Ltd., Cork, Ireland).

### Statistical Analysis

Patient characteristics are summarized with the mean and the standard deviation, and with numbers. Estimates for the study endpoints are presented with 95% confidence intervals. Positively skewed data such as ICU length of stay were logarithmically transformed, and are described with the geometric mean. To compare continuous and categorical data between the intervention and the control group, the two-sample t test and Fisher’s exact test were used, respectively. For the statistical testing of the changes in hemoglobin concentration from baseline over time we used an analysis of covariance (ANCOVA) [15]. The level of statistical significance was set at P<0.05. All analyses were performed with the JMP® 9.0 statistical software package (SAS Institute, Cary, NC, U.S.A.).

## Results

The control group consisted of 41 patients with a total of 617 observation days. In the BSB group 50 patients with a total of 559 observation days were included (Table 1). The baseline characteristics did not show any relevant differences.

**Table 1:** Baseline characteristics of the patients included in the study.

	Control (n = 41)	BSB (n = 50)
**Mean age**	62.1 (SD ± 16.1)	63.3 (SD ± 14.7)
**Male/female ratio**	24/17	34/16
**Mean SAPS II**	59.8 (SD ± 16.4)	62.5 (SD ± 15.7)
**Mean Hb on admission [g/dl]**	12.2 (SD ± 2.3)	12.8 (SD ± 1.8)

After the introduction and during the application of the BSB the mean blood loss per ICU day decreased from 43.3 ml (95% confidence interval (CI) 41.2–45.3 ml) in the controls to 15.0 ml (14.3–15.7 ml) in the BSB group (p < 0.001) as shown in Table 2. Generally, blood loss on ventilation days was higher compared to non-ventilation days. In detail, blood loss decreased from 48.7 ml (45.8–51.7 ml) in the controls to 16.1 ml (15.2–16.9 ml) in the BSB group (p < 0.001) on ventilation days, and blood loss on non-ventilation days was reduced from 35.1 ml (32.8–37.3 ml) in the controls to 11.1 ml (9.7–12.4 ml) in the BSB group (p < 0.001), indicating that blood loss reduction by BSB was achievable independently of the ventilation status. The median total ICU blood loss in the BSB-group was 145 ml (interquartile range [IQR] 96–206 ml) compared to 523 ml (IQR 360–911 ml, p < 0.001, U-test Mann-Whitney-Wilcoxon) in the control group.

**Table 2:** Average blood loss per Patient [ml] in controls compared to the BSB group per ICU day and during ventilation and non-ventilation days.

	Controls (n = 41 patients)		BSB (n = 50 patients)		P-value
	arithm.mean	95%- confidence interval	arithm.mean	95%- confidence interval	
**per ICU day [ml]**	43.3	41.2–45.3	15.0	14.3–15.7	<0.001
**per ventilation day [ml]**	48.7	45.8–51.7	16.1	15.2–16.9	<0.001
**per non-ventilation day [ml]**	35.1	32.8–37.3	11.1	9.7–12.4	<0.001

**Fig 1:**
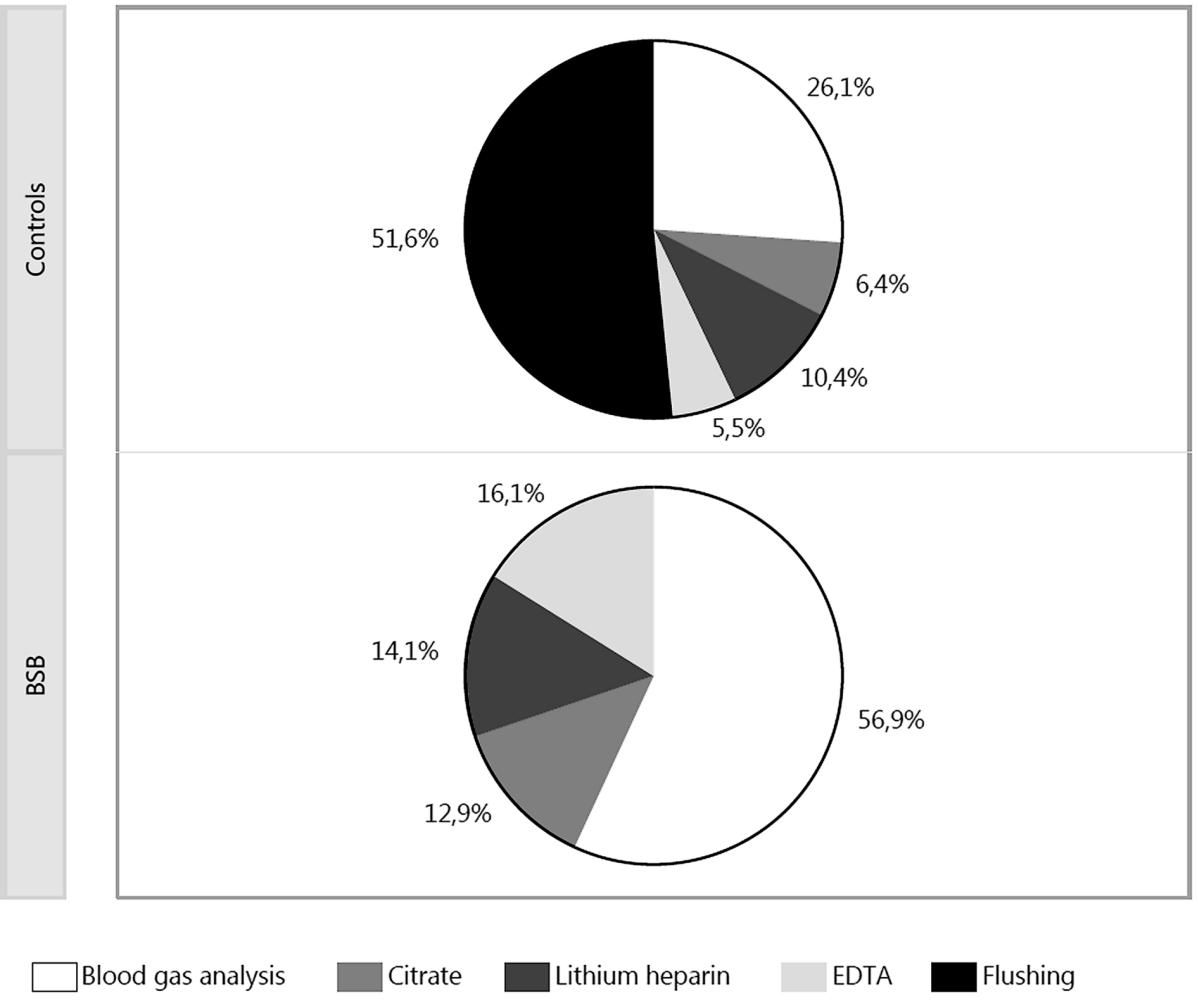
Percentage of blood loss in routine diagnostic blood samples characterized by the anticoagulant in the sampling tube (excluding blood cultures)

In the control group discarded blood used to flush the arterial line contributed to approx. 50% of the daily blood loss (Fig 1). On average, 2.6 ml of blood was discarded for every blood sampling to flush the line. Upon introduction of the BSB the number of blood gas tests was significantly reduced from 7.2 to 6.2 per ICU day (p = 0.002) and from 8.4 to 6.8 per ventilation day (p<0.001), respectively. Thus, a total flushing volume of 18.7 ml blood per day could be saved in the BSB group. Additionally, 9.7 ml blood per day could be saved by using smaller sized lithium-heparin tubes (2.7 ml instead of 5.5 ml) and a reduced filling of tubes and syringes. The total blood volume of the standard diagnostic blood sampling taken routinely at 5:00 a.m. (one arterial blood gas sample, one lithium-heparin tube, one citrate tube and one EDTA tube) +/- flushing volume was reduced from 13.3 ml to 7.3 ml (p<0.001) by the use of BSB.

**Fig 2:**
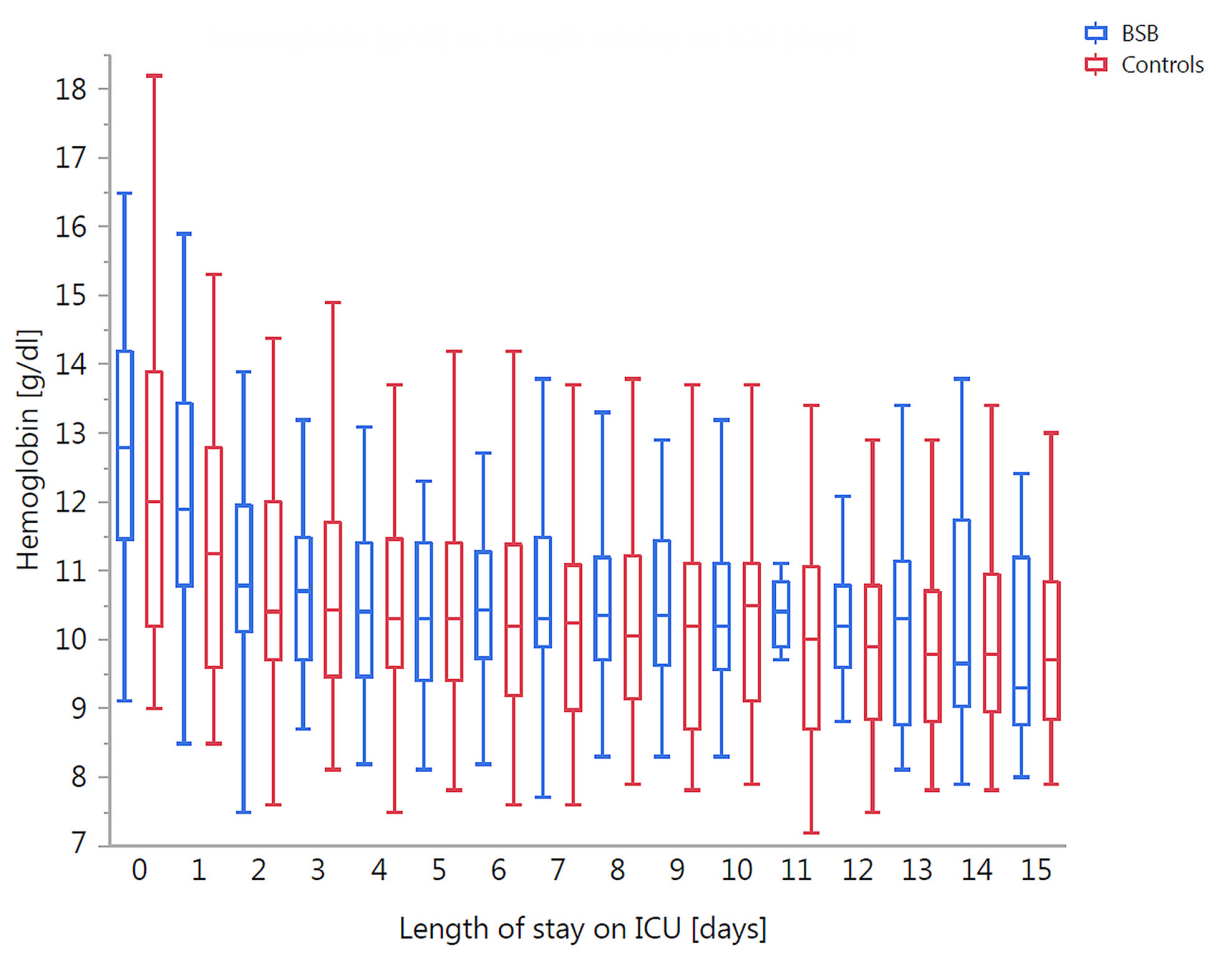
Box plots of the hemoglobin concentrations in the control and BSB group as a function of the length of stay in the ICU. (Note: The box shows the median with the 25^th^ and 75^th^ percentiles, and the whiskers indicate the minimum and maximum values.)

**Fig 3:**
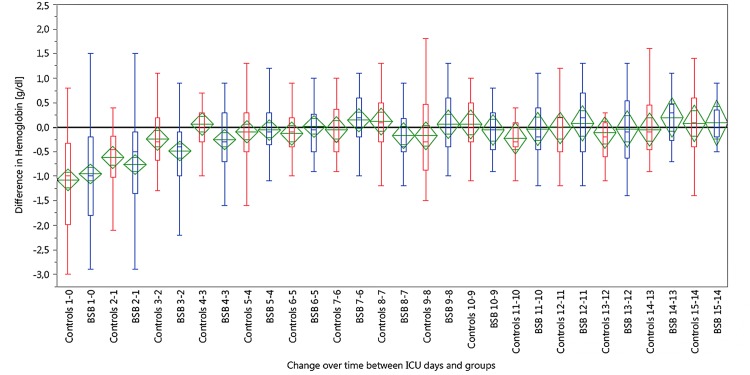
Box plots of the daily changes of the hemoglobin concentrations over time compared to the previous day in the control red) and in the BSB group (blue). (Note: The box shows the median with the 25th and 75th percentiles, and the whiskers indicate the minimum and maximum values. The mean diamonds (green) represent the mean and the 95% confidence interval of each group).

The mean hemoglobin value on the day of admission was 12.2 g/dl (11.5 to 12.9 g/dl) in the control group and 12.8 g/dl (12.3 to 13.4 g/dl) in the BSB group (p = ns). The distribution of hemoglobin values showed a similar decrease in both groups during the first 3 days of ICU stay and then reached a plateau around 10,5 g/dl (Figs 2 and 3). The hemoglobin values at day 5 and at ICU discharge were not significantly different in both groups (ANCOVA, supplemental data). The decrease in the mean hemoglobin concentration compared to the previous day was highest on day one after ICU admission and not affected by the BSB (Fig 3). However, hemoglobin values <8 g/dl and <9 g/dl were observed significantly fewer in the BSB group during the ICU stay (Table 3).

**Table 3:** Absolute number and percentage of observation days with hemoglobin concentrations < 8 g/dl and < 9 g/dl.

	Controls (n = 617 observation days)		BSB (n = 559 observation days)		P-value
	n (%)	95% confidence interval	n (%)	95% confidence interval	
**Hb < 8g/dl**	34 (5.5%)	3.9%-7.6%	13 (2.3%)	1.2%-3.9%	0.007
**Hb < 9g/dl**	131 (21.2%)	18.1%-24.7%	86 (15.4%)	12.5%-18.7%	0.010

In the control group 13 (31.7%) of the 41 patients received RBC transfusions in contrast to only 4 (8.0%) of the 50 patients in the BSB group (P = 0.006, Table 4). In the control group 23 transfusion events could be recorded with a total of 43 units of RBC transfused (7 units per 100 patient/days, Table 4). In contrast, in the BSB group only 7 transfusion events with a total of 13 RBC units (2.3 units per 100 patient/days) were necessary (P < 0.001). The hemoglobin concentration triggering the transfusion was not significantly different (8.2 vs. 7.8 g/dl).

**Table 4:** Blood transfusions.

	Controls (n = 41 patients)	BSB (n = 50 patients)	P-value
**Transfused patients**	13 (32%)	4 (8%)	0.006
**Units of transfused RBC**	43	13	—-
**Units of RBC per 100 patient days**	7.0	2.3	<0.001
**Transfusion events**	23	7	—-
**Transfusion events per 100 patient days**	3.7	1.3	0.003
**Mean Hb before transfusion [g/dl]**	8.2 (95% CI 7.9–8.5)	7.8 (95% CI 7.1–8.6)	ns

In the BSB-group we observed a mean ICU stay of 9.9 days vs. 13 days in the controls (p < 0.014, Table 5). The number of non-ventilation days after successful weaning in the BSB group was lower compared to the controls, whereas the number of days on mechanical ventilation was similar in both groups (Table 5). Mean total hospital stay was 21.1 days in the BSB group vs. 26.6 days in the controls (p = 0.04, Table 5).

**Table 5:** Mean ICU length of stay per patient in days, also separated in ventilation and non-ventilation days.

	Controls (n = 41 patients)		BSB (n = 50 patients)		P-value
	geom. mean	95%- confidence interval	geom. mean	95%- confidence interval	
**ICU days**	13.0	10.9–15.4	9.9	8.6–11.3	0.014
**- ventilation days**	7.1	6.1–8.3	7.5	6.6–8.5	ns
**- non-ventilation days**	4.6	3.4–6.1	2.5	1.9–3.3	0.004
**Total hospital stay**	26.6	22.3–31.8	21.1	18.4–24.3	0.04

## Discussion

In this study we could show that the introduction of the novel blood saving bundle (BSB) reduced the mean blood loss per ICU day by 65% from 43.3 ml to 15 ml. The most relevant component of the BSB was the introduction of a blood conservation device, whereas the smaller size of other blood sampling tubes and the reduced number of samples taken for blood gas analyses had only a minor effect.

The mean daily diagnostic blood loss in our patients before introduction of the BSB was in the same range as the 41 ml/day/patient reported in the ABC study [1]. In our study, about one half of the diagnostic blood loss was the discarded blood that was used to clear the arterial line from the crystalloid solution before taking the diagnostic blood sample. A volume of 2.6 ml discarded blood per sample and a mean of 7.2 samples for arterial blood gases per ICU day alone resulted in a daily blood loss of 18.7 ml, which could be saved solely by the introduction of a blood conservation device. Other studies have reported a volume of 8.3 to 22.4 ml discarded blood per day before the introduction of a blood conservation device [5,6,16–18].

The use of smaller test tubes achieved blood savings of < 3 ml per day. Higher savings would have been possible if we had switched to pediatric tubes [14,18]. However, we decided against pediatric tubes because they were not fully compatible with our automated laboratory analyzers and therefore would have delayed the laboratory diagnostics. In standard EDTA- and lithium-heparin tubes as well as in syringes for blood gas analysis underfilling occurred frequently possible, depending on the tests ordered. However, in citrate tubes for coagulation tests underfilling must be avoided because it leads to false test results.

For our study we selected a group of mechanically ventilated and critically ill intensive care patients with SAPS II-score of approximately 60. These patients routinely received an arterial line that besides arterial blood pressure monitoring, was also used for arterial blood gas analysis and other point-of-care laboratory tests such as blood glucose, lactate and electrolytes. We considered these patients at high risk for iatrogenic blood loss and development of anemia. Patients with other diseases that could contribute to anemia, such as hematologic diseases, severe renal failure or overt bleeding were excluded, as these additional variables would have hampered the interpretation of the data. Similar to most previous studies with blood conservation devices, we could not demonstrate an effect of the BSB on the mean Hb concentrations [5,16–19]. Both groups developed a mild anemia within the first three days of their ICU stay, which stabilized at values around 10.5 g/dl around day 4. This is in accordance with other studies that have postulated that anemia in ICU patients is a multifactorial process and that in many patients iatrogenic blood loss only contributes to about 20% of the total blood loss [4].

In the BSB group, however, fewer patients had Hb concentrations below 9 g/dl or 8 g/dl and received significantly less RBC transfusions. The mean Hb concentration before transfusion was 7.8 g/dl in the BSB group vs. 8.2 g/dl in the controls, which was not significantly different. Notably, in both study periods we applied the same transfusion policy with a standard transfusion trigger of 7.0 g/dl. The decision to transfuse a patient at higher Hb values was made on an individual basis with patients with e.g. hemodynamic instability, chronic obstructive pulmonary disease or difficult weaning. However, due to the longitudinal study design, we cannot exclude a bias that might have interfered with the multi factorial based decision to transfuse a patient. In a recent study by Mukhopadhyay et al. the use of a blood conservation device together with a restrictive transfusion strategy (Hb threshold 7.5 g/dl) significantly reduced transfusions from 0.131 units/patient/day in the controls to 0.068 units/patient/day (p = 0.02) [13]. The corresponding values in our study were 0.070 units/patient/day in the controls vs. 0.023 units/patient/day in the BSB group. It could be demonstrated t that in effect we were more restrictive in our transfusion policy. In a subgroup analysis of the study by Mukhopadhyay et al. blood conservation devices had no effects on transfusion requirements in low-risk patients with preserved hemoglobin concentrations. Vice versa it has been speculated that high risk ICU patients with renal failure and/or primary bleeding, which were frequently excluded from trials with blood conservation devices including our own study, might benefit the most from blood conservation devices [5,13,20].

In our study we observed a shorter ICU length of stay in the BSB group. As the number of days on mechanical ventilation was similar in both groups, the shorter ICU stay in the BSB group was due to less non-ventilation days in the ICU after successful weaning. The total hospital stay also was shorter in the BSB group. However, these findings have to be interpreted with extreme caution because of the longitudinal study design. We cannot exclude changes in practice pattern, imbalances of patient characteristics or disease severity or other unrecognized confounding factors that might have influenced earlier transfer to the general ward or an earlier discharge from the hospital. A detailed analysis of other factors that might explain a shorter ICU length of stay in the BSB patients, however, was beyond the scope of this study. Previous studies provided evidence that blood transfusions can have a negative impact on the treatment course of ICU patients including increased mortality [21,22]. In the study by Mukhopadhyay et al. the use of a blood conservation device was associated with a reduced mortality, but not with a reduced length of stay in the ICU [13]. In our study, we cannot provide information about the impact of the BSB on mortality, because patients who died in the ICU were excluded from the study.

By the end of the study, the BSB was successfully implemented into the clinical routine in our ICU. We agree with authors of previous studies that blood conservation devices are easy to use, and that additional costs are negligible and might be more than compensated by reduced transfusion requirements [5]. In clinical routine we did not observe any confirmed arterial line infections, which is also in line with former studies [5].

## Conclusion

The present study shows that a closed-loop arterial blood sampling system was the most effective tool to reduce diagnostic blood loss in mechanical ventilated ICU-patients and might reduce the need for RBC transfusions in these patients at high risk for developing anemia under a restrictive transfusion regimen.

### Key Messages

- The implementation of the BSB could significantly reduce blood loss during ICU stay.
- A closed-loop arterial blood sampling system contributed most to the reduction of blood loss, whereas smaller tubes and a reduction of blood gas analysis frequency had only a minor effect.
- The implementation of the BSB might reduce RBC transfusion requirements in mechanically ventilated critically ill patients.

## Supporting Information

S1 Dataset (XLS)

## Abbreviations

BSB: blood-saving bundle
Hb: hemoglobin
RBC: red blood cell
ICU: intensive care unit
SD: standard deviation
EDTA: ethylenediaminetetraacetic acid
SAPS II: Simplified Acute Physiology Score II
